# Food Hubs as a Means to Promote Food Security in Post-Secondary Institutions: A Scoping Review

**DOI:** 10.3390/nu14193951

**Published:** 2022-09-23

**Authors:** Rachel A. Murphy, Yu Jacqueline Guo, Heloise Sierra Melo Pinto Cordeiro, Sumara Stroshein, Casey Hamilton, Sara Kozicky

**Affiliations:** 1 School of Population and Public Health, University of British Columbia, 2206 East Mall, Vancouver, BC V6T 1Z3, Canada; 2 Cancer Control Research, BC Cancer, 610 W 10th Ave, Vancouver, BC V5Z 1L3, Canada; 3 UBC Wellbeing, University of British Columbia, 2260 West Mall, Vancouver, BC V6T 1Z4, Canada; 4 Campus Health, University of British Columbia Okanagan, 3272 University Way, Kelowna, BC V1V 1V7, Canada

**Keywords:** food hub, food security, food systems, sustainability

## Abstract

An estimated 20 to 50% of post-secondary students experience food insecurity. Students who are food insecure are more likely to have poor health and lower academic performance relative to food secure peers. Food hubs are physical or digital spaces that provide access to food initiatives and wraparound programs such as employment placement or income support are increasingly of interest as a means to respond to food insecurity. We conducted a scoping review to identify best practices and effective approaches to food hubs that promote food security in post-secondary institutions in North America. The Medline, Embase, CAB Direct and Web of Science databases were searched. A total of 4637 articles were identified and screened by two reviewers. Four articles were included. They encompassed a mix of interventions: a campus pantry and garden, a food rescue program, food literacy-based curriculum and a toolkit to support implementation of interventions on campus. The heterogeneity of studies precluded identification of best practices, but positive impacts of all interventions were noted on metrics such as self-efficacy and greater awareness of food insecurity. The gap in evidence on effective approaches that promote campus food security is a critical barrier to development and implementation of interventions, and should be addressed in future studies.

## 1. Introduction

Food insecurity; limited physical, social and economic access to sufficient, safe, and nutritious foods that meet food preferences and dietary needs [1], is a rising public health problem among students attending post-secondary institutions; education proceeding high school instruction including universities, colleges and institutes. Estimates suggest that 20% to more than 50% of post-secondary students experience food insecurity [2,3,4], a rate that is three to four times higher than the general population [5]. The higher rates of food insecurity reflect a combination of factors. Enrollment trends reflect growing numbers of students from lower socioeconomic backgrounds, greater racial and ethnic diversity and record numbers of international students [6] who are more likely to experience food insecurity. The growing cost of living, high cost of a post-secondary education, insufficient financial aid/bursaries, greater financial hardship among low to middle income families, and in some countries like the United States, exclusion of some post-secondary students from programs such as the Supplemental Nutritional Assistance Program further add to the higher prevalence of food insecurity [4]. Students who experience food insecurity are more likely to have poorer health including diabetes, obesity, depression and overall self-rated health [7,8]. Students who experience food insecurity are also more likely to have poorer academic outcomes; lower grades, delayed graduation and higher likelihood of dropping out [8,9,10]. The growing recognition of food insecurity at post-secondary institutions and the broader implications, has led to interest in strategies for effective, robust and sustainable approaches to diversify supports for students who are burdened with food insecurity on post-secondary campuses.

The creation of food banks or food pantries in the community and at post-secondary institutions is among the most common responses to addressing food insecurity [11]. Food banks are spaces where donated and/or purchased groceries can be accessed for no cost by individuals and families. However, while food banks play an important role in immediate access to food, evidence has shown that they have limited effect on improving overall food security in the community [12] and on post-secondary campuses [13]. Food banks focus on the provision of foods versus addressing the root cause of food insecurity—income, and evidence suggests that use of food banks is one of the least common strategies used by severely food-insecure households when met with financial challenges [14]. Food banks have been criticized for a limited ability to meet individuals’ food needs and not providing access to foods in a dignified, socially acceptable manner [15,16]. Income and housing policies are critical to promote food security, but in the absence of systems level changes, alternative food initiatives (AFIs) such as community gardens, cooking skill development programs, community kitchens, farmers markets, food waste ‘rescue’ programs, low-cost food markets and food budgeting among others are potential means to empower individuals and lessen the burden of food insecurity [17]. One criticism of AFIs, however, is inadequate engagement of individuals most at risk of food insecurity; low income, racialized, marginalized and other vulnerable populations in shaping the initiatives [18,19].

The term ‘food hub’ has been increasingly adopted to describe a gathering place (physical or digital) that serves as a foundation for sustainable food systems. Although food hubs may differ based on communities’ needs, they generally encompass multiple AFIs that provide access to food, food literacy and wellness programming in combination with wraparound programs (e.g., employment services, enrolment in public benefits). Food hubs may consider food banks as one element rather than the totality of the response, and thus can create a more effective, dignified and sustainable solution to food insecurity. Food hubs can serve as a space to create connections between community members and provide opportunities for community engagement. Although promising, food hubs are a relatively new approach to building a dignified, sustainable food security system. The best means to accomplish this is thus unclear and may differ depending on the population and setting of respective interventions.

This scoping review was conducted in parallel to a community (staff, students and faculty) based participatory action research process at the University of British Columbia Vancouver (UBC-V) campus. The collective goal was to identify best practices and effective approaches to inform the development and implementation of a food hub to help lessen the burden of community food insecurity on campus. The specific objective of this scoping review was to systematically identify existing interventions or approaches to food hubs in post-secondary institutions in North America. The research question was: what is known from the literature about best practices and efficacy of food hubs or similar models that promote campus food security? The findings may have applicability to our work at UBC-V in addition to broader relevance to post-secondary institutions who are considering how to respond to food insecurity on campus.

## 2. Materials and Methods

The PRISMA extension for scoping reviews checklist was used to inform the conduct and reporting of the scoping review including defining the population of interest, search and data extraction strategies. A reference librarian at UBC developed the scoping review search strategy which used four databases: Medline (Ovid), Embase (Ovid), CAB Direct and Web of Science. MeSH terms and keywords included variations of the terms “food security”, “food supply”, “food or cooking”, “universities” and “students” in the title, subject headings, abstract, keywords or full text. See Supplementary Materials Table S1 for details. The search strategy was executed by the study team (SS, YJG and HSMPC). All search results were exported from the respective database and imported into Covidence (Melbourne, Australia) for selection and screening.

Articles were included in the scoping review if they met the following inclusion criteria; higher education setting, published in the past ten years (2011 to 2021), and described an intervention or summary of AFIs and/or a food hub to address food security. Exclusion criteria were food hubs that focused on distribution of local foods and farmers revenue, community food hubs (i.e., those not in a post-secondary institution setting), setting outside of North America, focus on dietary/nutrition assessment or food safety, food security initiatives related to children/pediatrics/elementary/middle/high school, manuscripts with a sole focus on emergency food supply models (e.g., food banks or food pantries), or manuscripts not published in English.

Two reviewers (SS and RAM) separately screened all identified titles and abstracts to select manuscripts that fit inclusion/exclusion criteria. Agreement for the initial screen was 80%. Those with differing opinions were reviewed, discussed and consensus was reached. Both reviewers also conducted the full text screening of articles that were included from the title and abstract screening. Agreement for the full text review was 95%. After discussion, the one article in conflict was subsequently excluded. Following selection, SS abstracted information on the location of the study, timeframe, study design, target population and characteristics as well as key findings, strengths and limitations from each manuscript as applicable.

## 3. Results

**Figure 1:**
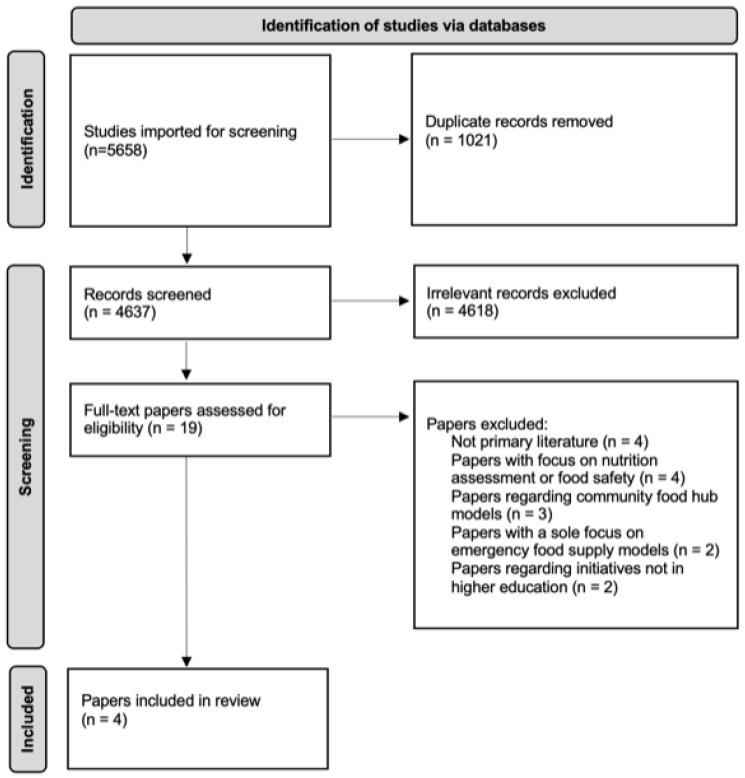
Overview of the study identification and screening process.

A total of 4637 studies were screened for title and abstract (Figure 1). After screening the title and abstracts, 4618 articles did not meet the inclusion/exclusion criteria. Full text review was subsequently performed for 19 articles. Of these, four were excluded as they were not primary studies, four were focused on nutrition assessment or food safety, three studied community-based food hubs, two were focused on emergency food provision and two did not take place in a post-secondary institution. This resulted in four studies being selected for inclusion in the scoping review.

All the studies were based at post-secondary institutions in the United States. The studies were diverse in design spanning one case study [20], one pre-post study over a 7 month period [21], a program summary [22], and a cross-sectional study [23]. The timeframe of the studies ranged from a single timepoint. None of the studies included a food hub. Rather, one study (Frank et al. [22]) focused on the evaluation of a singular AFI—a food rescue program—while Ullevig et al. [20] and Morgan et al. [21] focused on multiple AFIs—a community garden and pantry in Ullevig et al. and food literacy curriculum spanning efficacy and cooking skills in Morgan et al. One study (Hagedorn et al. [23] described the development of a toolkit that may facilitate implementation of multiple AFIs, although not specifically within a food hub context. The three studies that included student participants differed with respect to demographics, including primarily Caucasian participants (92%) in Morgan et al. [21], to primarily Hispanic (37%) and African American (22%) participants in Ullevig et al. [20]. Food insecurity was prevalent in all three studies of students, from a ‘low’ of 28% [20] to a high of 59% [21]. Additional details of the included studies can be found in Table 1.

All of the studies noted success with respective study outcomes, although the outcomes were too disparate to identify common successes or facilitators of success (Table 2). On an individual study level, key takeaways included the need to involve staff to limit the impact of student turnover within AFIs as well as increased awareness of the prevalence of food insecurity and importance of sustainability in Ullevig et al. [20]. The food rescue program [22] and food literacy-based curriculum [21] both noted positive experiences of participants in the studies. For example, positive broader impacts in the pilot study by Frank et al. [22] including reduced waste of food and normalization of food rescue. Other particularly noteworthy successes include the simple and budget friendly nature of the food rescue online program that the authors noted would facilitate scale up [22], as well as improvements in food literacy based self-efficacy and confidence in cooking and food preparation skills after just 11-weeks [21]. Based on the findings from Hagedorn et al. [23], attention to layout, content and initiatives/programs in a toolkit to inform implementation of AFIs, is critical for acceptance by stakeholders who would lead said implementation.

All of the studies identified barriers and limitations to success of the programs and the broader goals of achieving food security. For instance, Ullevig et al. reported [20] low awareness of the intervention (community garden and food pantry) and limited access to refrigeration which confined the type of food donations. The food rescue program noted that some participants reported that their experience with the program was awkward, food was difficult to find or had run out. They were also unable to quantify whether food waste was actually reduced (not a planned measurable), or the impact of the program on student hunger, food insecurity and other aspects of health, well-being and academic performance [22]. Morgan et al. [21] noted that despite improvements in food literacy skills, there was no improvement in food security. Although the toolkit developed by Hagedorn et al. [23] was well-received by stakeholders, they identified barriers to implementation, namely the need to strengthen the evidence base on food security initiatives on post-secondary campuses. In particular, little research has been published that provide replicable methods for implementation and evaluation of student food insecurity initiatives.

## 4. Discussion

This scoping review aimed to provide insight into best practices and effective approaches to inform the development and implementation of a food hub to promote community food security on post-secondary campuses. However, the existing published evidence in the field is very limited. We did not identify any studies that included a food hub (physical or digital) or similar structure. Overall, only four studies met our inclusion criteria, which were variable in design, objectives and evaluation. It was thus not possible to identify best practices. Rather, the identified studies targeted different aspects of food insecurity (i.e., food literacy, food skills and access), different populations (i.e., stakeholders and students) as well as a diversity of approaches (i.e., a mobile tool, in-classroom curriculum), which collectively could form a food hub. In particular, consideration of dedicated staff in the development and sustainability of a food hub, use of mobile tools to support initiatives to help facilitate reach and scalability and in-class time for delivery of AFIs may help facilitate uptake by students. Additionally, the importance of de-stigmatizing food insecurity and AFIs, and the need for increased awareness of AFIs were mentioned as common facilitators of success across the disparate studies. We therefore suggest the planning process of food hub initiatives should consider how to address these critical issues, including potentially developing a social marketing plan as in Ullevig et al. [20].

The limitations identified within the studies are also informative to consider in the development of food hubs at post-secondary institutes. The student populations captured in the studies were relatively limited, and may therefore not have captured the overall demographics or experiences of those who engaged in the respective programs. For instance, participants in Morgan et al. [21] were predominately Caucasian while the prevalence of food insecurity in Ullevig et al. [20] was lower than other demographic estimates at other post-secondary institutes [2,3]. The design of the studies generally precluded the ability to measure the effectiveness of interventions as only one of the studies (Morgan et al. [21]) used a pre and post design, and found no impact of the curriculum-based intervention on food insecurity, although positive impacts on self-efficacy and food skills were observed. Given that the root cause of food insecurity is income, it is perhaps unsurprising that a curriculum-based food literacy and skills program delivered over a short timeframe did not impact this metric. Rather, it reinforces the need for establishment of food hubs that encompass AFIs that deliver food literacy and skills programming together with wraparound services to more comprehensively support options to help people manage food insecurity.

There are several limitations to this scoping review that should be acknowledged. We were specifically interested in identifying AFIs and/or food hubs that could inform the development of a food hub at UBC-V in Canada. As such, we confined the search criteria to higher education institutes in North America due to perceived similarity of student populations and campus environments. However, this may have contributed to the limited number of studies identified. It is also possible that differences between institutions or student populations in the four identified studies relative to our setting at UBC-V and other institutions may limit the transferability of evidence. For example, the identified studies were a mix of private and public post-secondary institutions of moderate size (e.g., <30,000 students) and included students who were predominately Caucasian, Hispanic or African American. In contrast, there are nearly 60,000 students at UBC-V, of which, 27.2% are international students, predominately from East Asia and South Asia [24]. This scoping review was also focused on published literature. It is possible that consideration of ‘grey’ literature may have captured additional studies on AFIs or food hubs that have not been published in academic journals. Although a prior (unpublished) environmental scan of grey literature by our team only identified one food hub at a Canadian post-secondary institution that would meet the inclusion/exclusion criterion in this review.

All of the studies included in this scoping review occurred before the COVID-19 pandemic, and as such, considerations of approaches to support food security that do not rely on in-person initiatives was absent. The COVID-19 pandemic has resulted in increased food insecurity in Canada [25], as well as shifts to remote instruction in education and as a result, fewer students present on campuses. Public health measures have at times also restricted the ability to provide programming of some AFIs such as community meals or food skills. Although, it is unclear what the future impact of the COVID-19 pandemic and resultant public health measures will be, it may be pertinent to consider flexibility in the delivery of programmatic implementation and potential surges in demand for food hubs and related services.

## 5. Conclusions

Overall, the studies included in this scoping review suggest positive impacts of diverse programs targeting food insecurity among students at post-secondary institutions. Approaches such as campus pantries and gardens, food rescue programs, classroom-based food education and toolkits to support planning and implementation may help to meet community needs and diversify support options that are not stigmatizing to lessen the burden of food insecurity. However, the general lack of evidence on which AFI approaches are the most effective, acceptable and sustainable in post-secondary institutions is a major gap that impedes identification of best practices and is a barrier to implementation. The small number of studies identified in this review was particularly striking given the comparatively large body of evidence on food hubs and AFIs in community settings [26,27]. Future research that describes processes related to developing, implementing and evaluating on-campus food security initiatives is critical to supporting broader institutional initiatives to improve food security among students.

## Figures and Tables

**Table 1:** Characteristics of included studies.

Authors	Location	Timeframe of Study	Target Population	Participant Characteristics	Study Design
Ullevig et al. [20]	University of Texas at San Antonio	2017–2018	Students attending an urban institution	Survey of 438 students: 37% Hispanic, 22% African American, 15% Caucasian, 9% Multi-racial, 8% Asian, 9.6% Other/Unknown, 46% First-generation students, 7% had dependents, 28% were food insecure	Case Study
Frank et al. [22]	La Salle University	Not reported	All students	Focus group of 38 undergraduate nutrition students. Survey of 206 students: 74% on-campus residents, 78% employed at least part-time, 36% food insecure	Program summary
Morgan et al. [21]	Appalachian State University	2019	Students in a food science laboratory	Survey of 51 students: 92% Caucasian, 59% female, 47% Sophomore year, 66% lived off campus, 55% were not employed, 59% reported high food security at pre-assessment	Pre-post
Hagedorn et al. [23]	Multiple institutes in the United States	Not reported	Stakeholders from post-secondary institutes	30 stakeholders from 27 institutions completed a survey: 87% female, mean age 41 years, mean of 11.5 years in their profession	Cross-sectional

**Table 2:** Key findings of included studies.

Authors	Objective	Key Findings
Ullevig et al. [20]	To describe lessons learned from the establishment of a community garden and food pantry	Several challenges were identified including high turnover of students and volunteers, lack of awareness of the garden/pantry, lack of capacity for fresh food donations, limited variety of foods offered. Successes included staff involvement for continuity, increased awareness of food insecurity and sustainability within the institution in part due to the social marketing plan
Frank [22]	To describe experiences and perspectives of students who participated in a pilot of an online program to distribute free food that would otherwise be thrown away	Over 12 months, 451 students enrolled in the pilot program with increasing engagement overtime. Reduced waste of catered foods, normalization of food rescue. The online program was effective, simple and budget friendly.
Morgan et al. [21]	To implement a food literacy-based curriculum to increase food literacy-based skills and self-efficacy and reduce food insecurity among students enrolled in an established Food Science Laboratory course	Improvements were observed for food literacy-based behaviors, food literacy based self-efficacy and confidence in cooking and food preparation skills. Overall positive experiences in the program. No change in food security was observed (59% high food security at pre-assessment versus 63% at post-assessment).
Hagedorn et al. [23]	To develop a toolkit for improving food security at higher education institutions based on a literature review and evaluate the toolkit among stakeholders	The toolkit included recommendations on implementation of food pantries, campus gardens, farmers markets, dining and recovery program, mobile applications and policy change. The toolkit was highly rated with respect to layout, content and initiatives/programs included but 50% identified barriers to implementation of the toolkit.

## Data Availability

All data used in this review are available in cited published literature.

## Supplementary Materials

The following supporting information can be downloaded at: https://www.mdpi.com/article/10.3390/nu14193951/s1, Table S1: Scoping Literature Review Strategy.
