# Supplementary material for: Food Hubs as a Means to Promote Food Security in Post-Secondary Institutions: A Scoping Review
Source: Nutrients. 2022 Sep 23;14(19):3951. doi: 10.3390/nu14193951 (PMC9572345; doi:10.3390/nu14193951)
Supplement: Supplementary file 1 [file nutrients-14-03951-s001.zip › nutrients-1920828-supplementary.pdf]

Supplementary Table S1. Scoping Literature Review search strategy

**Date Searched**	**Database**	**Search Strategy**
June 11, 2021	MEDLINE (Ovid)	1 food supply/ or food deserts/ or food insecurity/ or food security/ 14044<br>2 (food security or food insecurit* or food sovereignty).mp. [mp=title, abstract, original title, name of substance word, subject heading word, floating sub-heading word, keyword heading word, organism supplementary concept word, protocol supplementary concept word, rare disease supplementary concept word, unique identifier, synonyms] 11601<br>3 ((food or cooking) adj3 (hub* or network* or strateg* or program* or movement* or desert* or gathering* or center* or workshop* or sharing or communit*)).mp. [mp=title, abstract, original title, name of substance word, subject heading word, floating sub-heading word, keyword heading word, organism supplementary concept word, protocol supplementary concept word, rare disease supplementary concept word, unique identifier, synonyms] 7719<br>4 1 or 2 or 3 27531<br>5 Universities/ 44047<br>6 exp Students/ 138341<br>7 (universit* or college* or student* or undergraduate* or post secondary or higher education or campus).mp. [mp=title, abstract, original title, name of substance word, subject heading word, floating sub-heading word, keyword heading word, organism supplementary concept word, protocol supplementary concept word, rare disease supplementary concept word, unique identifier, synonyms] 796511<br>8 5 or 6 or 7 796511<br>9 4 and 8 1185
June 11, 2021	CAB Direct	(ab:((food) near/1 (hub* or network* or strateg* or program* or movement* or desert* or gathering* or center* or workshop* or sharing or communit*)) AND ab:(universit* or college* or student* or undergraduate* or "post secondary" or "higher education" or campus))
June 11, 2021	CAB Direct	(("community engagement") AND ((food) near/1 (hub* or network* or strateg* or program* or movement* or gathering* or center* or workshop* or communit*)) AND (universit* or college* or student* or undergraduate* or "post secondary" or "higher education" or campus))
June 11, 2021	Embase (Ovid)	1 food supply/ or food deserts/ or food insecurity/ or food security/ 26537<br>2 (food security or food insecurit* or food sovereignty).mp. [mp=title, abstract, heading word, drug trade name, original title, device manufacturer, drug manufacturer, device trade name, keyword, floating subheading word, candidate term word] 15317<br>3 ((food or cooking) adj3 (hub* or network* or strateg* or program* or movement* or desert* or gathering* or center* or workshop* or sharing or communit*)).mp. [mp=title, abstract, heading word, drug trade name,

		original title, device manufacturer, drug manufacturer, device trade name, keyword, floating subheading word, candidate term word] 9466<br>4 1 or 2 or 3 38485<br>5 (universit* or college* or student* or undergraduate* or post secondary or higher education or campus).mp. [mp=title, abstract, heading word, drug trade name, original title, device manufacturer, drug manufacturer, device trade name, keyword, floating subheading word, candidate term word] 1464279<br>6 4 and 5 3179
June 11, 2021	Web of Science	((ALL=("food security" or "food insecurit*" or "food sovereignty")) AND (ALL=(universit* or college* or student* or undergraduate* or "post secondary" or "higher education" or campus)) AND TS=((food) NEAR/1 (hub* or network* or strateg* or program* or movement* or desert* or gathering* or center* or workshop* or sharing or communit*)))
